# Supplementary material for: Mechanisms governing the pioneering and redistribution capabilities of the non-classical pioneer PU.1
Source: Nat Commun. 2020 Jan 21;11:402. doi: 10.1038/s41467-019-13960-2 (PMC6972792; doi:10.1038/s41467-019-13960-2)
Supplement: Supplementary file 7 — Source data [file 41467_2019_13960_MOESM7_ESM.zip › Source_Data/Figure6/Figure6B_MotifScanOutput/ETS1_mutPU.1/homerResults/motif32.similar.html]

motif32

## Information for motif32

C
G
A
T
C
A
T
G
C
G
T
A
A
G
T
C
T
C
A
G
G
C
A
T
G
A
T
C
G
T
C
A
A
C
G
T
G
A
T
C
  
Reverse Opposite:  

C
T
A
G
T
G
C
A
A
C
G
T
C
A
T
G
C
G
T
A
A
G
T
C
C
T
A
G
G
C
A
T
G
T
A
C
C
G
T
A
  

|  |  |
| --- | --- |
| p-value: | 1e-53 |
| log p-value: | -1.230e+02 |
| Information Content per bp: | 1.678 |
| Number of Target Sequences with motif | 921.0 |
| Percentage of Target Sequences with motif | 6.83% |
| Number of Background Sequences with motif | 1221.0 |
| Percentage of Background Sequences with motif | 3.48% |
| Average Position of motif in Targets | 103.2 +/- 55.9bp |
| Average Position of motif in Background | 101.3 +/- 58.6bp |
| Strand Bias (log2 ratio + to - strand density) | -0.0 |
| Multiplicity (# of sites on avg that occur together) | 1.02 |
| Motif File: | file (matrix) reverse opposite |

### Similar de novo motifs found

|  |  |  |  |  |  |  |  |
| --- | --- | --- | --- | --- | --- | --- | --- |
| Rank | Match Score | Redundant Motif | P-value | log P-value | % of Targets | % of Background | Motif file |
| 1 | 0.908 | T C A G T G C A G A C T A C T G T G C A A T G C T C A G C G A T G T A C T C G A A C G T | 1e-22 | -52.950766 | 1.56% | 0.57% | motif file (matrix) |
| 2 | 0.877 | G A T C G T A C T C A G C T G A C G A T A C T G C T G A A G T C A C T G G A C T G T A C G C T A | 1e-16 | -38.304466 | 1.01% | 0.35% | motif file (matrix) |
